# Comparison of *tet*(X4)-containing contigs assembled from metagenomic sequencing data with plasmid sequences of isolates from a cohort of healthy subjects

**DOI:** 10.1128/spectrum.03969-23

**Published:** 2024-03-05

**Authors:** Yichen Ding, Shuan Er, Abel Tan, Jean-Sebastien Gounot, Woei-Yuh Saw, Linda Wei Lin Tan, Yik Ying Teo, Niranjan Nagarajan, Henning Seedorf

**Affiliations:** 1 Temasek Life Sciences Laboratory, 1 Research Link, Singapore, Singapore; 2 Genome Institute of Singapore (GIS), Agency for Science, Technology and Research (A*STAR), Singapore, Singapore; 3 Baker Heart and Diabetes Institute, Melbourne, Victoria, Singapore; 4 Saw Swee Hock School of Public Health, National University of Singapore, Singapore, Singapore; 5 NUS Graduate School for Integrative Science and Engineering, National University of Singapore, Singapore, Singapore; 6 Department of Statistics and Applied Probability, National University of Singapore, Singapore, Singapore; 7 Life Sciences Institute, National University of Singapore, Singapore, Singapore; 8 Department of Biological Sciences, National University of Singapore, Singapore, Singapore; University of Saskatchewan, Saskatoon, Canada

**Keywords:** tigecycline, *tet*X4, fecal microbiota, metagenomics, cultivation, *Enterobacteriaceae*, florfenicol, tylosin

## Abstract

Recently discovered *tet*(X) gene variants have provided new insights into microbial antibiotic resistance mechanisms and their potential consequences for public health. This study focused on detection, analysis, and characterization of Tet(X4)-positive Enterobacterales from the gut microbiota of a healthy cohort of individuals in Singapore using cultivation-dependent and cultivation-independent approaches. Twelve Tet(X4)-positive Enterobacterales strains that were previously obtained from the cohort were fully genome-sequenced and comparatively analyzed. A metagenomic sequencing (MS) data set of the same samples was mined for contigs that harbored the *tet*(X4) resistance gene. The sequences of *tet*(X4)-containing contigs and plasmids sequences were compared. The presence of the resistance genes *floR* and *estT* (previously annotated as *catD*) was detected in the same cassette in 10 and 12 out of the 12 *tet*(X4)-carrying plasmids, respectively. MS detected *tet*(X4)-containing contigs in 2 out of the 109 subjects, while cultivation-dependent analysis previously reported a prevalence of 10.1%. The *tet*(X4)-containing sequences assembled from MS data are relatively short (~14 to 33 kb) but show high similarity to the respective plasmid sequences of the isolates. Our findings show that MS can complement efforts in the surveillance of antibiotic resistance genes for clinical samples, while it has a lower sensitivity than a cultivation-based method when the target organism has a low abundance. Further optimization is required if MS is to be utilized in antibiotic resistance surveillance.

**IMPORTANCE:**

The global rise in antibiotic resistance makes it necessary to develop and apply new approaches to detect and monitor the prevalence of antibiotic resistance genes in human populations. In this regard, of particular interest are resistances against last-resort antibiotics, such as tigecycline. In this study, we show that metagenomic sequencing can help to detect high abundance of the tigecycline resistance gene *tet*(X4) in fecal samples from a cohort of healthy human subjects. However, cultivation-based approaches currently remain the most reliable and cost-effective method for detection of antibiotic-resistant bacteria.

## INTRODUCTION

Genomic surveillance is one important measure to monitor and control the spread of multidrug resistant bacteria (MDR) in the human population. In general, the prevalence for MDR bacteria is determined through a cultivation-dependent approach; i.e., clinical or environmental samples are inoculated into selective agar plates supplemented with antibiotics to obtain MDR bacterial colonies, which will be confirmed with phenotypic and molecular assays. Selected colonies can be further subjected to whole-genome sequencing and comparative genomic analysis to determine their antibiotic resistance genes, plasmid sequences, and possible transmission clusters. This approach has allowed detailed insights into the genomic structure of many MDR bacteria by numerous studies, especially for the recently emerged *tet*(X) family-mediated tigecycline-resistant Enterobacterales (1, 2). On the other hand, shotgun metagenomic sequencing (MS) characterizes the microbial communities in clinical and environmental samples via an unbiased culture-independent approach in which the total DNA of the samples is extracted and sequenced. Subsequently, the antibiotic resistance genes of interest can be further analyzed using the contigs assembled from metagenomic sequencing data to determine their presence in the samples, as well as the associated plasmid types and host species. Shotgun metagenomic sequencing may therefore complement culture-based whole-genome sequencing approaches for rapid identification of MDR bacteria, especially when cultivation of bacteria is difficult, or a high-throughput screen is required.

The recently identified *tet*(X) family tigecycline resistance genes are variants of the initially described *tet*(X) (3). These new variants share 95% sequence identity with *tet*(X), which also confers high-level resistance to last-resort antibiotics such as tigecycline (4), posing a serious threat to public health (5). Among the various emerging *tet*(X) variants, *tet*(X4) has been identified in animals, healthy individuals, and patients in multiple provinces of China and other regions (6), and its successful transmission could be attributed to conjugative plasmids and *IS*CR2-mediated transposition (7). We previously reported that the prevalence of Tet(X4)-producing Enterobacterales in the gut microbiota of healthy individuals in Singapore is 10.1% and analyzed the sequences of two IncI1-type plasmids (p2EC1-1 and p94EC-2) that carry *tet*(X4) (1). Here, we further sequenced and characterized additional 12 tigecycline-resistant Enterobacterales strains isolated from human fecal samples in Singapore. We show that *tet*(X4) is associated with a diverse range of plasmid types and hosts and is potentially co-transferred with florfenicol resistance gene *floR* and tylosin resistance gene *estT*. The latter has only recently been characterized as a serine-dependent macrolide esterase (8, 9). We further leveraged on recently published high-quality metagenomic sequence data for the same fecal samples collected from the cohort to evaluate if contigs assembled from metagenomic sequencing data could reveal *tet*(X4) plasmid sequences (1, 10). Our findings suggest that metagenomic sequencing could complement culture-based surveillance for MDR bacteria if they are present at high abundance in clinical samples.

## MATERIALS AND METHODS

### Sample collection and DNA extraction

The collection and DNA extraction of fecal samples have been described previously (1, 10). In brief, feces from 109 individuals aged 48–76  years old of the Singapore Integrative Omics Study were collected in 2018 using a BioCollector (BioCollective) kit, according to the manufacturer’s instructions. Fecal samples were handled in a Coy anaerobic chamber containing N_2_ (75%), CO_2_ (20%), and H_2_ (5%) gas mixture. Homogenized samples were transferred to 50-mL screw-cap tubes prior to storage at –80°C. The QIAamp Power Fecal Pro DNA kit was used to extract gDNA for genomic (2 × 2 mL pure culture, OD_600_ = 0.17) and metagenomic (fecal material, ~0.5 g) sequencing. DNA for genomic sequencing was further purified using a Qiagen Genomic-tip 20/G kit as described in the manufacturer’s protocol (Qiagen, Germany). Cells from cultures were concentrated at 10,000 × *g* for 15 min before DNA extraction. DNA was quantified using a Qubit v.1.0 fluorometer with a broad-range assay kit (Life Technologies) and a NanoDrop-2000 (Thermo Fisher Scientific).

### Genome sequencing and data analysis

Genomic DNA of previously isolated strains was extracted using Qiagen Genomic-tip 20/G as per manufacturer’s instructions. Whole-genome sequencing was performed using MinION and Illumina Novaseq, followed by genome assembly and polishing using Flye v.2.9 (11, 12) and Pilon v.1.24 (13), respectively. The assembled complete genomes were subjected to sequence typing by online MLST v.2.0 (14), phylogenetic analysis using the Harvest Suite (15), antibiotic resistance gene prediction by ResFinder v.4.1 (16), plasmid typing by PlasmidFinder v.2.0 (17), and identification of insertion sequences by ISFinder (18). Comparative sequence analysis was performed using EasyFig v.2.2.5 (19) running BLAST+ v.2.13.0 (20).

### Metagenomic sequencing assembly and analysis

MS contigs are derived from the Singapore Platinum Metagenomes Project (SPMP) (10), which was conducted on DNA extracted from the same fecal samples that were also used for the cultivation-based analysis. Contigs containing the *tet*(X4) gene were identified using BLAST, and subsequent verification was performed using ResFinder with default settings (16, 20).

### CFU counting

Colony-forming unit (CFU) counting experiment was done for our previous study (1). Briefly, frozen fecal samples were weighed and inoculated into Luria broth, followed by incubation at 37°C with 200-rpm shaking for 3 h. The fecal suspensions were then serially diluted in 0.9% NaCl and spotted onto MacConkey agar plates supplemented with 2-mg/L eravacycline dihydrochloride. The CFUs were enumerated after incubation at 37°C for 18 h, and the results were normalized to CFU per gram of input fecal sample.

### GenBank accession numbers

MS short and long reads can be found under BioProject number PRJEB49168, and genomes sequences can be found under BioProject number PRJNA599529.

## RESULTS AND DISCUSSION

### Characterization of *tet*(X4)-carrying plasmids by whole-genome sequencing

**Fig 1:**
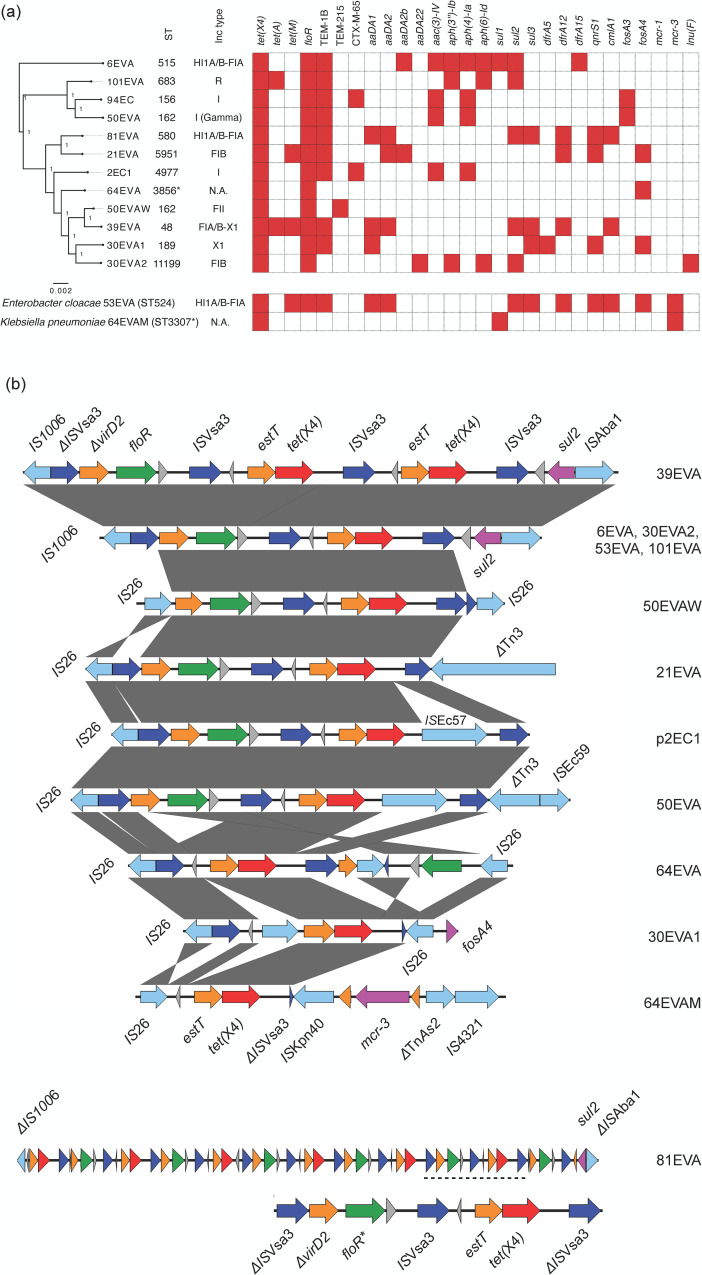
(a) Antibiotic resistance gene profiling and phylogenetic analyses of *tet*(X4)-positive Enterobacterales strains. The phylogeny, sequence type, and incompatibility group of the *tet*(X4)-harboring plasmids are shown in the figure (N.A. indicates the plasmid is not typeable). The heat map shows the antibiotic resistance genes carried by *tet*(X4)-carrying plasmid. The presence of antibiotic resistance genes is indicated by red blocks. (b) Comparison of *tet*(X4) genetic environments. Open reading frames and their directions of transcription are indicated by colored arrows. Red: *tet*(X4); blue: *IS*Vsa3 (corresponding to *IS*CR2); green: *floR*; magenta: other antibiotic resistance genes; light blue: mobile genetic elements and transposases; orange: genes with putative functions; gray: hypothetical proteins. Truncated genes are indicated by the symbol Δ, whereas *flo*R contains a mutation indicated by an asterisk (*) for isolate 81EVA.

Twelve Enterobacterales strains that are positive for *tet*(X4) were previously isolated from human fecal samples on MacConkey agar plates supplemented with eravacycline (1). Their genomes have been sequenced to complete-genome level by Illumina and Nanopore. In total, *tet*(X4) was carried by seven different plasmid types, including IncHI1A/B-IncFIA (*n* = 3), IncFIB (*n* = 2), IncI (gamma, *n* = 1), IncX1 (*n* = 1), IncFIA/B-IncI (*n* = 1), IncFII (*n* = 1), and IncR (*n* = 1), while two plasmids were non-typable (Fig. 1a). The host bacterial species include *Escherichia coli* (*n* = 10), *Klebsiella pneumoniae* (*n* = 1, isolate 64EVAM, ST3307*), and *Enterobacter cloacae* (*n* = 1, isolate 53EVA, ST524) (Fig. 1a). In particular, the 10 *tet*(X4)-positive *E. coli* strains belonged to 10 different sequence types (Fig. 1a). These results suggested that a broad range of *E. coli* strains with diverse genetic backgrounds had been associated with *tet*(X4) in Singapore, which is consistent with findings previously reported in other regions such as China, Thailand, and Pakistan (6, 7, 21).

We further characterized and compared the genetic environments of *tet*(X4) among the 10 plasmids (Fig. 1b). We also included two previously reported isolates, 2EC1 and 94EC, which carried *tet*(X4) and *bla*_CTX-M-65_ in our comparative genomic analysis (1). It was found that *tet*(X4) is closely associated with *IS*Vsa3 by having at least one copy of *IS*Vsa3 at its upstream, except for 64EVAM. This is consistent with previous studies showing *tet*(X4) is probably mobilized via *IS*Vsa3 (*IS*CR2)-mediated transposition (1, 2). In addition, we found that other resistance genes are co-occurring with *tet(X4*); i.e., the *estT* is found in all 12 cassettes, while florfenicol resistance gene *floR* is located in the same cassette in 10 out of the 12 *tet*(X4)-carrying plasmids (Fig. 1b), including Δ*IS*Vsa3-Δ*virD2-floR-IS*Vsa3-*estT-tet*(X4)-*IS*Vsa3 (39EVA, 6EVA, 30EVA2, 53EVA, 101EVA, and 81EVA); Δ*IS*Vsa3-Δ*virD2-floR-IS*Vsa3-*estT-tet*(X4)-*IS*EC57-*IS*Vsa3 (50EVA); *IS*26-Δ*virD2-floR-IS*Vsa3-*estT-tet*(X4)-*IS*Vsa3 (21EVA and 50EVAW); and *IS*26-*IS*Vsa3-*estT-tet*(X4)-*IS*Vsa3-orf-*IS*26-*floR-IS*26 (64EVA). For 30EVA1 and 64EVAM, although *floR* and *tet*(X4) are not in the same cassette, *floR* was either on the same plasmid as *tet*(X4) in 30EVA1 or carried by another plasmid in 64EVAM (Fig. 1a). Such close association was not found for other antibiotic resistance genes identified in the 12 strains (Fig. 1). The *floR* gene could confer resistance to florfenicol and chloramphenicol, while *estT* confers resistance against 16-membered ring-containing macrolide antibiotics, including tylosin, tilmicosin, and tildipirosin (22–24). Most of these antibiotics are commonly used as veterinary medicine in aquaculture, swine, cattle, and poultry (23, 25–27) . Similarly, the emergence of *tet*(X4) and other *tet*(X) variants was suggested to be related to the overuse of tetracycline in the food industry in China (2). However, Singapore lacks primary food industry and imports most of the agriculture products from other countries. The co-carriage of *floR*, *estT*, and *tet*(X4) by MDR plasmids isolated in healthy individuals in Singapore suggested that their origin might be linked to importation of animal products from other countries (28, 29). Nonetheless, we could not rule out that the emergence of *tet*(X4)-carrying MDR plasmids in Singapore could also arise due to the rampant inappropriate use of antimicrobials in various other sectors, while further studies should be carried out to track their origin.

### Evaluation of shotgun metagenomic sequencing in detection of *tet*(X4)-carrying plasmids

**Fig 2:**
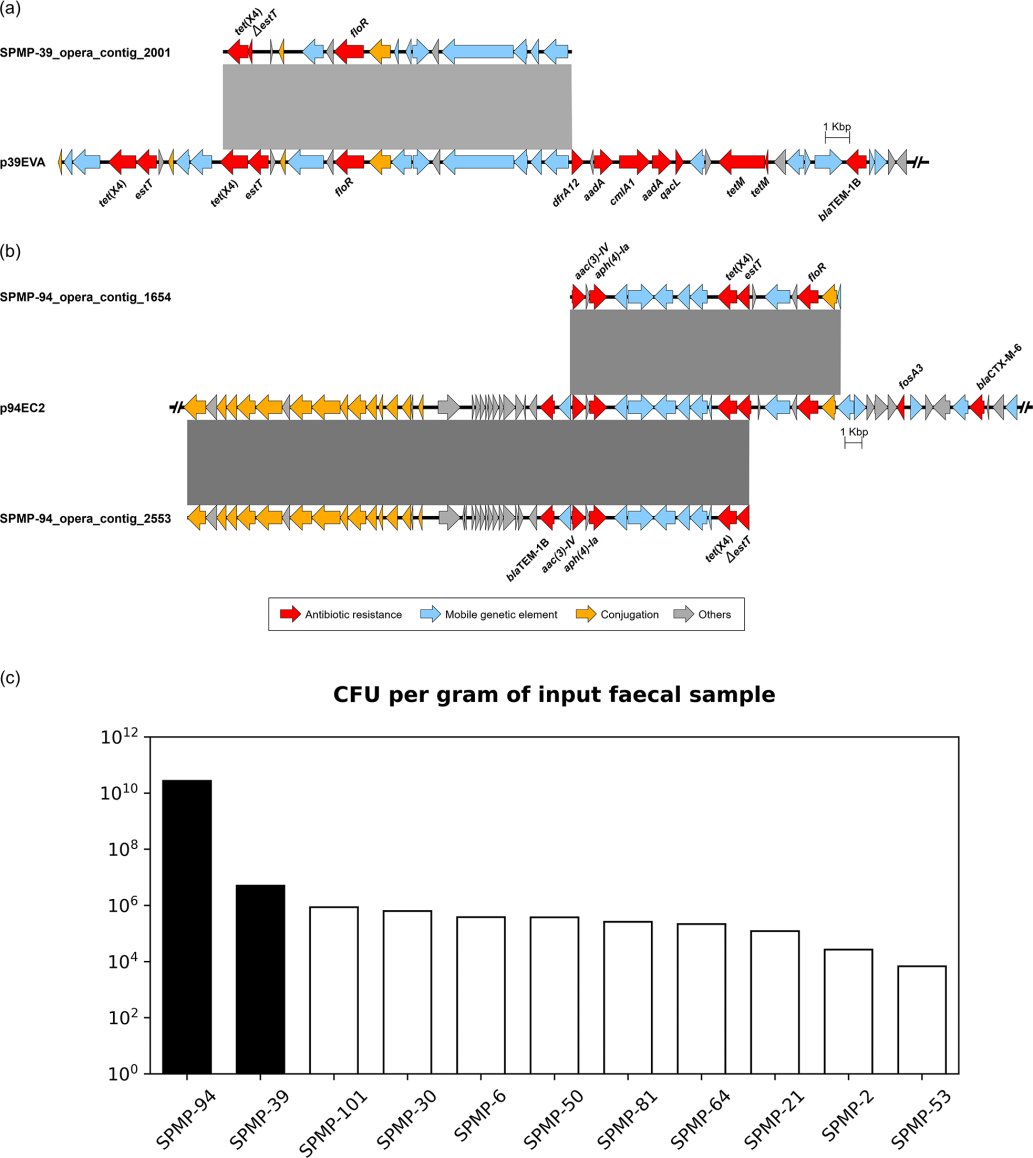
Comparative analysis between metagenomic sequencing-assembled contigs and plasmid sequences containing *tet*(X4) genes derived from (a) SPMP-39 and (b) SPMP-94. Genes and their respective transcriptional directions are represented by arrows with colors indicating their functional classifications. Shown are truncated sequences of the plasmids. Truncated genes are indicated by the symbol Δ. The gray-shaded rectangles indicate regions (>4,500 bp) of (a) 98.8% and (b) >99.5% homology. (c) Colony-forming unit counts of *tet*(X4)-positive Enterobacterales from fecal samples. Contigs assembled from shotgun metagenomic sequencing data contained *tet*(X4)-carrying contigs for subjects SPMP-39 and SPMP-94 (black bars) but not for the other fecal samples (white bars). Of note, the fecal samples were incubated in lysogeny broth prior to inoculation onto selective agar plate (see Materials and Methods). This is to allow the *tet*(X4)-positive Enterobacterales to recover from frozen stock before being exposed to the antibiotic for accurate CFU counting. All fecal samples were incubated under the same conditions for the same period of time.

In total, 11 fecal samples contain *tet*(X4)-positive Enterobacterales, and the *tet*(X4)-carrying plasmid sequences were analyzed in this study (Fig. 1) and in our previous study (1). To assess if shotgun metagenomic sequencing could detect *tet*(X4)-carrying plasmids, we further screened the contigs assembled from shotgun metagenomic sequencing for *tet*(X4). Interestingly, we found that *tet*(X4)-harboring contigs can only be detected in two fecal samples (subject SPMP-39 and SPMP-94). The sizes of the *tet*(X4)-harboring contigs (14–33 kbp) were shorter than the plasmids (101–134 kbp). A comparison of the *tet*(X4)-harboring contigs with the plasmid sequences revealed high homology of the contigs to the plasmid sequences (Fig. 2). This finding indicates that shotgun metagenomic sequencing may potentially aid in the detection of *tet*(X4) and its surrounding genetic environment.

Enterobacterales is often present in low abundance in the human gut, which may potentially result in lower sensitivity for the detection of its associated antibiotic resistance genes when using shotgun metagenomic sequencing. We therefore wondered if the detection of *tet*(X4)-carrying contigs from shotgun metagenomic sequencing data is related to the abundance of the *tet*(X4)-positive Enterobacterales in the fecal samples. Interestingly, out of the three *tet*(X4)-harboring contigs identified, two were detected in subject SPMP-94, who uncoincidentally has a much higher CFU count—by four orders of magnitude—than subject SPMP-39 and the other nine samples for which MS failed to detect *tet*(X4)-containing contigs (Fig. 2c). Thus, these results suggest that shotgun metagenomic sequencing could detect *tet*(X4)-harboring plasmids when the bacteria containing the plasmid are present in high abundance in clinical samples.

Taken together, we report that *tet*(X4) is associated with a broad range of plasmids and host bacteria in the gut of healthy Singaporeans and is closely associated with florfenicol resistance gene *floR* and tylosin resistance gene *estT*. By comparing the contigs assembled from shotgun metagenomic sequencing, we show that this approach could complement culture-based detection of *tet*(X4) plasmids in human fecal samples when present at higher abundance. Further optimization is required if metagenomic sequencing should be used to discover MDR from clinical and environmental samples. However, selective cultivation currently remains the most reliable and cost-effective approach for detection of antibiotic-resistant bacteria.
